# Prediction of Knee Prosthesis Using Patient Gender and BMI With Non-marked X-Ray by Deep Learning

**DOI:** 10.3389/fsurg.2022.798761

**Published:** 2022-03-14

**Authors:** Yu Yue, Qiaochu Gao, Minwei Zhao, Dou Li, Hua Tian

**Affiliations:** ^1^Department of Electronics, Peking University, Beijing, China; ^2^Department of Orthopedics, Peking University Third Hospital, and Engineering Research Center of Bone and Joint Precision Medicine, Ministry of Education, Beijing, China

**Keywords:** total knee arthroplasty, prosthesis prediction, deep learning, error correct output coding, transfer learning

## Abstract

**Background:**

Total knee arthroplasty (TKA) is effective for severe osteoarthritis and other related diseases. Accurate prosthesis prediction is a crucial factor for improving clinical outcomes and patient satisfaction after TKA. Current studies mainly focus on conventional manual template measurements, which are inconvenient and inefficient.

**Methods:**

In this article, we utilize convolutional neural networks to analyze a multimodal patient data and design a system that helps doctors choose prostheses for TKA. To alleviate the problems of insufficient data and uneven distribution of labels, research on model structure, loss function and transfer learning is carried out. Algorithm optimization based on error correct output coding (ECOC) is implemented to further boost the performance.

**Results:**

The experimental results show the ECOC-based model reaches prediction accuracies of 88.23% and 86.27% for femoral components and tibial components, respectively.

**Conclusions:**

The results verify that the ECOC-based model for prosthesis prediction in TKA is feasible and outperforms existing methods, which is of great significance for templating.

## Introduction

Total knee arthroplasty (TKA) is an effective surgical intervention for treating end-stage osteoarthritis, and promising clinical outcomes have been reported worldwide (1). However, due to a lack of attention to accurate preoperative prosthesis prediction, patients' postoperative satisfaction with TKA has been approximately 80% (2). Having an accurate prosthesis template before surgery is a vital factor in achieving satisfactory clinical outcomes and reducing medical costs.

Previous studies of templating systems mainly include physical X-ray templates and digital template measurements (3). The former uses a physical template ruler provided by the prosthesis manufacturer to perform manual measurements on the X-ray images to select an appropriate prosthesis. The latter refers to the use of a digital template measurement system to match preoperative X-ray images with possible prosthetic types. Both methods require rescaled X-ray films based on a reference ball to set a consistent amplification rate for real joints. The accuracy of measurement varies randomly according to the quality of the radiographs. Moreover, template measurement is labor-intensive and relies heavily on the experience of doctors. Therefore, template measurements are prone to increase the risk of prosthesis mismatch in TKA and lead to further complications (4).

In the post-5G era, research on 6G technology is increasing, and intelligent medicine is attracting much more attention. Artificial intelligence-aided medicine is an expanding area, and it is anticipated to provide a better user experience. Deep learning technology has already been applied to the medical field and demonstrated its powerful modeling capabilities (5). In orthopedics, the application of deep learning mainly focuses on the diagnosis of fractures and osteoarthritis (6–8). Because knee prosthesis selection in TKA has a clear operating criterion and internal logic, it can be well-modeled with deep learning approaches, we establish a convolutional neural network (CNN)-based prediction system to achieve automatic and accurate prosthesis prediction.

First, in this work, we propose and construct a novel deep learning model for prosthesis size selection with knee radiographs and patient physical information as input. To enhance the accuracy of prediction, classic image processing techniques such as contrast-limited adaptive histogram equalization (CLAHE) (9) and mean-shift (10) are used as preprocessing methods to remove redundant radiograph information. Refinements to the loss function and model structure are implemented to address the problem of uneven distribution of labels. Transfer learning (11) is also introduced to compensate for data deficiencies.

To further enhance the performance of the prediction model, we optimize the prediction algorithm for component selection in TKA based on error correct output coding (ECOC) (12). An appropriate error control coding scheme and coding length are determined considering accuracy and computational complexity. Different decision rules for mapping model outputs to prosthesis sizes are studied to employ the optimal one.

The results of the experiments validate that a CNN is effective in processing a patient's information and making an accurate prediction. ECOC can provide other benefits as well. The final performance of the predictive model exceeds that of manual measurement by medical experts.

The remainder of the article is organized as follows. Section Basic Principle gives a brief introduction to related principles comprising error control coding, distance metrics and ECOC-based multiclassification models. The prediction platform based on CNN is depicted in Section Methods and is organized as the implementation of the basic predictive model and ECOC-based optimization. Section Results reports the data analysis and experimental results. Discussion of the results is presented in Section Discussion.

## Basic Principle

### Error Control Coding

The concept of error control coding was initially applied in telecommunication. The demand for efficient and reliable data transmission and storage systems has been accelerated by the emergence of large-scale, high-speed data networks for exchange, processing and so on. A major concern of the system design is the control of errors so that the data from transmitters can be reliably reproduced at receivers (13). In this article, we mainly deal with block codes, one of the commonly used error control coding schemes, to fulfill our task.

A message block in block coding is represented by the binary k-tuple **u** = (*u*_0_, *u*_1_, …, *u*_*k*−1_). A binary encoder transforms each message **u** independently into a binary n-tuple **v** = (*v*_0_, *v*_1_, …, *v*_*n*−1_) of discrete symbols called a codeword. To have a different codeword assigned to each message, *k* < *n* is ensured. The *n*−*k* redundant bits provide a code that can be used to correct errors (13).

Hamming codes and Hadamard codes are two important linear block codes. For any positive integer *m*≥3, there exists a Hamming code with the following parameters:

- Code length *n* = 2^*m*^−1
- Number of information symbols *k* = *n*−*m*
- Number of parity-check symbols *m*.

The codewords of a Hadamard code consist of the row vectors of Hadamard matrices. An n-dimensional Hadamard matrix has the following features (14):

- *n* is an even number, and all elements in the matrix take the value of either 0 or 1.
- One line of the matrix is all 0s, and the rest of the lines contain *n*/2 0s and *n*/2 1s.
- The Hamming distance between any two lines is *n*/2.

We can obtain *M*_2*n*_ from *M*_*n*_ with Equation (1), where $\overset{\bar{}}{M_{n}}$ represents the complement matrix of *M*_*n*_.


(1) $$\{\begin{array}{c} M_{2}=\left[\begin{array}{cc} 0 & 0\\ 0 & 1 \end{array}\right]\\ M_{2n}=\left[\begin{array}{cc} M_{n} & M_{n}\\ M_{n} & M_{n} \end{array}\right] \end{array}$$

### Distance Metrics

*Hamming distance* is one of the widely adopted distance metrics for evaluating the error-correcting performance of different codes. For two n-tuples **w** and **v**, the Hamming distance between them, denoted by *d*_*H*_(*w, v*), is defined as the number of positi ons where the components differ. Given a block code C, the minimum distance *d*_*H, min*_ is


(2) $$\begin{array}{r} {\text{d}}_{\text{H},\text{min}}≜\mathrm{mtextn}\left\{{\text{d}}_{\text{H}}\left(\text{w},\text{v}\right):\text{w},\text{v}\in \text{C},\text{w}\neq \text{v}\right\} \end{array}$$

*d*_*H,min*_ determines the random-error-correcting capabilities of a code. Code C with *d*_*H,min*_ can correct all the error patterns with *t* or fewer bits, where *t* is a positive integer meeting the condition of 2*t*+1 ≤ *d*_min_ ≤ 2*t*+ 2.

If *d*_*H, min*_ of a Hamming code is 3, it can only correct one error, regardless of how long the code is. For a Hadamard code, *d*_*H, min*_ = *n*/2, and its correcting capability is in accordance with half the code length. Thus, longer Hadamard codes have better error correcting performance.

The Hamming distance can be used in hard decision decoding by comparing the similarity of received vectors with different codewords at receivers. In addition to Hamming distances, *Euclidean distance* is also popularly employed in similarity-based decoding, which is referred to as soft decision decoding.

The Euclidean distance between two vectors **v** and **w** in n-tuple space is defined as:


(3) $$\begin{array}{r} Euclidean\left(\text{v},\text{w}\right)=\sqrt{\overset{n}{\underset{i=1}{\sum }}{\left(\text{v}\left[i\right]-\text{w}\left[i\right]\right)}^{2}} \end{array}$$

It can be inferred that Euclidean distance contains more specific difference information than Hamming distance.

### ECOC-Based Classification

In general, there are two main approaches to multiclass classification: direct multiclass representation and decomposition design (15). The former aims to design multiclass classifiers directly, while the latter tries to split the original task into multiple binary subproblems.

For decomposition design, there are many approaches, such as one-vs.-all (16), one- vs.-one (17) and ECOC-based algorithms (12). Dietterich and Bakiri (15) proposed a binary ECOC framework comprised of three steps:

1. **Encoding**. In this stage, a coding matrix is determined, and each class is represented by one row of the matrix.
2. **Binary classifier learning**. The dataset of each classifier depends on the corresponding column of the coding matrix.
3. **Decoding**. A specific class is predicted based on the output sequence of the classifiers and the method of decoding.

In this article, we choose the Hamming code and the Hadamard code as the optimum schemes to boost the accuracy of neural networks. The number of output nodes in networks needs to be compatible with codewords. We will study the effects of coding schemes, code lengths, decision rules, etc., on the accuracy of classification.

## Methods

### Participants

This study has been approved by Peking University Third Hospital Medical Science Research Ethics Committee. Information on patients diagnosed with knee osteoarthritis and undergoing TKA was collected to constitute the dataset.

We selected patients who take the primary total knee arthroplasty in Peking University Third Hospital from 2018 to 2020. The selected dataset includes the X-rays of knees before and after the operation, where domestic AK posterior stable knee prosthesis are applied. As a training set, only cases with satisfied prothesis size would be included for deep learning model. Therefore, cases meet the following conditions are excluded:

1. The prothesis overhangs or the lack of coverage is more than 2 mm.
2. Prosthesis malalignment.
3. Femoral notching.
4. With long stem or screw.

### Overall System Design

**Figure 1:**
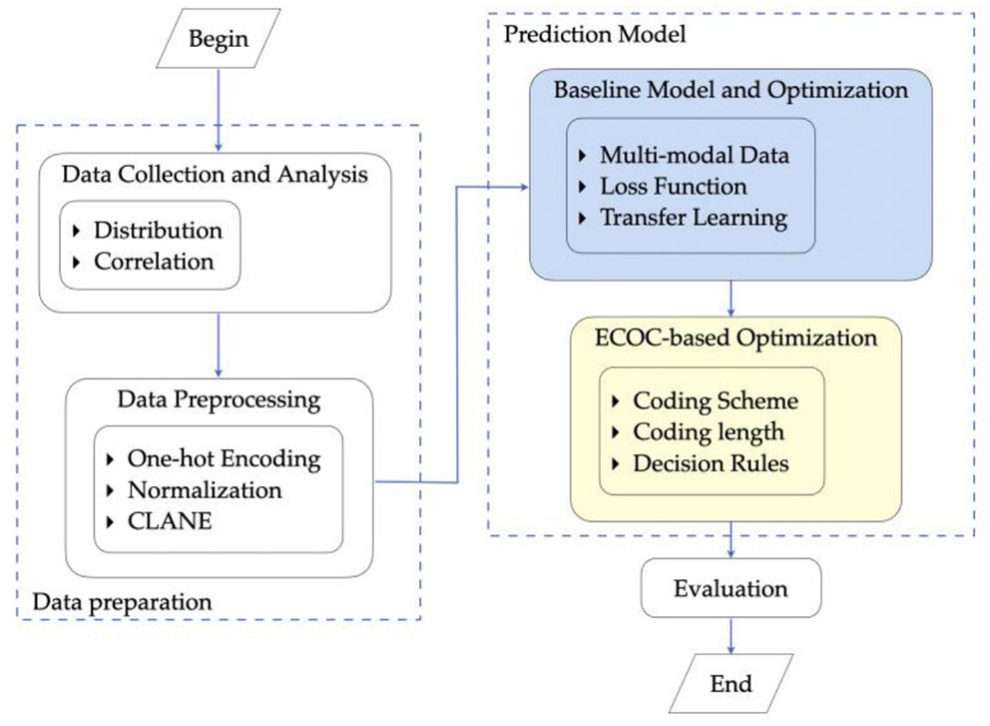
Research procedure.

As Figure 1 shows, the research procedure of the proposed prediction system for prosthesis selection mainly consists of data preparation, implementation and optimization of the predictive model, and evaluation.

Specifically, first, we complete the process of data collection. The anterior-posterior and lateral X-ray images of the patients' knees together with physical information are utilized as training features. The radiographs provide primary information, and physical information provides supplementary features for model training. Abnormal data are excluded from the final dataset in this step. Furthermore, the distribution of data and the correlation between features are analyzed as the analytics foundation for further modeling.

Then, preprocessing is employed on the prepared dataset to remove noise. This step benefits the training of CNN models. Following that, a CNN model is exploited. Optimization of the model structure and loss function along with transfer learning is further carried out to achieve better prediction performance.

To construct a more accurate prediction model, data are preprocessed according to their types. For the categorical feature gender, one-hot encoding is conducted. For continuous values, such as height and weight features, normalization is applied.

For X-ray images, redundant information such as text information and noise are removed through CLAHE (9), mean-shift (10) and cropping methods. After these operations, the images are ready as proper input for model training. Processed by the prediction model, the predicted prosthesis sizes are compared with the practically used ones and calculated a prediction accuracy.

### Baseline Predictive Modeling

A baseline model for prediction is constructed based on (18) and introduced briefly in this section. To address the vanishing gradient problem in deep learning, a deep residual learning framework, ResNet (19), is introduced and selected as the fundamental structure for our prediction platform. A commonly used 18-layer ResNet framework is adopted in consideration of both the computational complexity and prediction accuracy.

**Figure 2:**
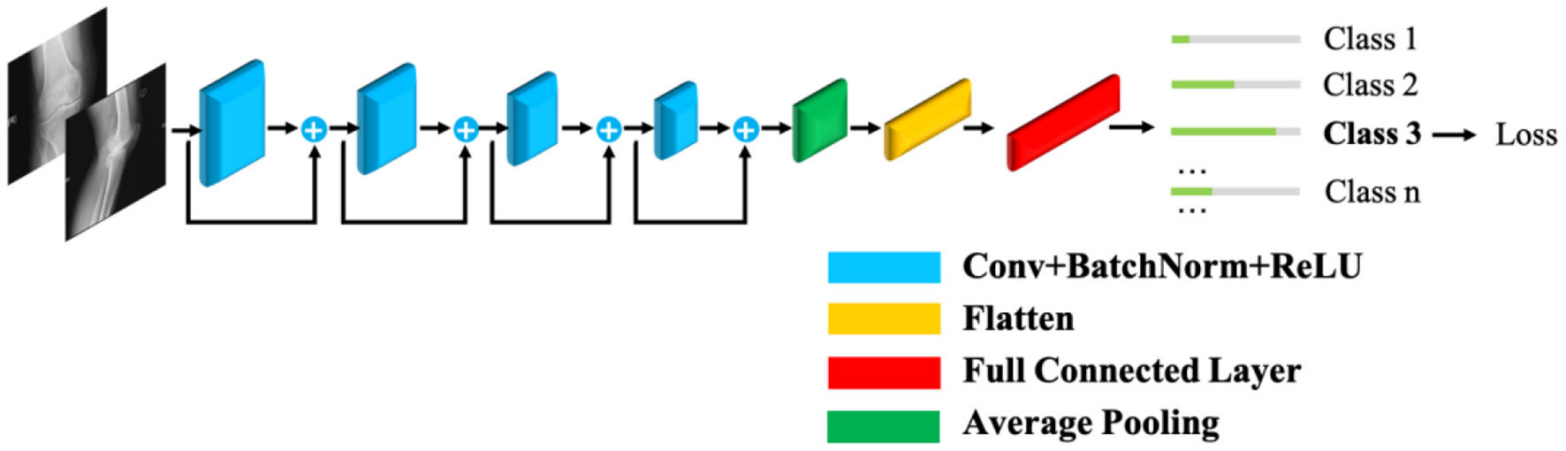
The distribution of prosthetic size by gender.

The ResNet18 structure is illustrated in Figure 2. The radiographs are fed sequentially into the network. Features are extracted by four groups of convolutional layers. Finally, fully connected layers output the prediction. This configuration is hereinafter referred to as the baseline model.

To address the problem of uneven distribution, a focal loss function (20) based on cross-entropy is introduced. The refined loss function is defined in Equation (4), where *f*_*i*_ denotes the output of a fully connected layer, *p*_*i*_ is the predicted probability, *N*_*i*_ represents the number of types *i*, and β and γ are hyperparameters to be tuned.


(4) $$\begin{array}{r} \frac{L_{i}=-\alpha_{i}{\left(1-P_{i}\right)}^{\beta }\log\left(P_{i}\right)}{\begin{array}{c} P_{i}=e^{f_{i}}/\sum_{j}e^{f_{j}}\\ \alpha_{i}={\left(1/N_{i}\right)}^{\gamma }/\sum_{j}{\left(1/N_{j}\right)}^{\gamma } \end{array}} \end{array}$$

Along with cross-entropy loss, two additional factors, α_*i*_ and ${\left(1-p_{i}\right)}^{\beta }$, are considered. The former enables models to pay more attention to classes with fewer samples, and the latter focuses more on hard-to-train classes.

Inspired by Zhang et al. (21), an automatic fine-grained recognition approach is introduced to amend the inadequacy of samples in each class, which flexibly responds to any object annotation at both the training and testing stages.

Moreover, for the problem of data deficiency, transfer learning is applied. The model is pretrained with the MURA dataset (22) and then fine-tuned to accommodate our knees dataset.

### ECOC-Based Optimization

The ECOC-based prediction model introduces error control coding to decompose the multiclass classification into several binary subtasks and enable error correction. To utilize the relationships among different binary classifiers, we employ an 18-layer ResNet (19) to train these binary tasks jointly. Each output node of the CNN represents a binary classifier, and its value represents the probability of the positive class of the corresponding classifier.

To further enhance prediction performance, coding specifications such as coding scheme, length and decision rules are explored to find an appropriate set according to the prediction accuracy and other criteria, e.g., computational complexity, and the optimal setting is selected.

**Figure 3:**
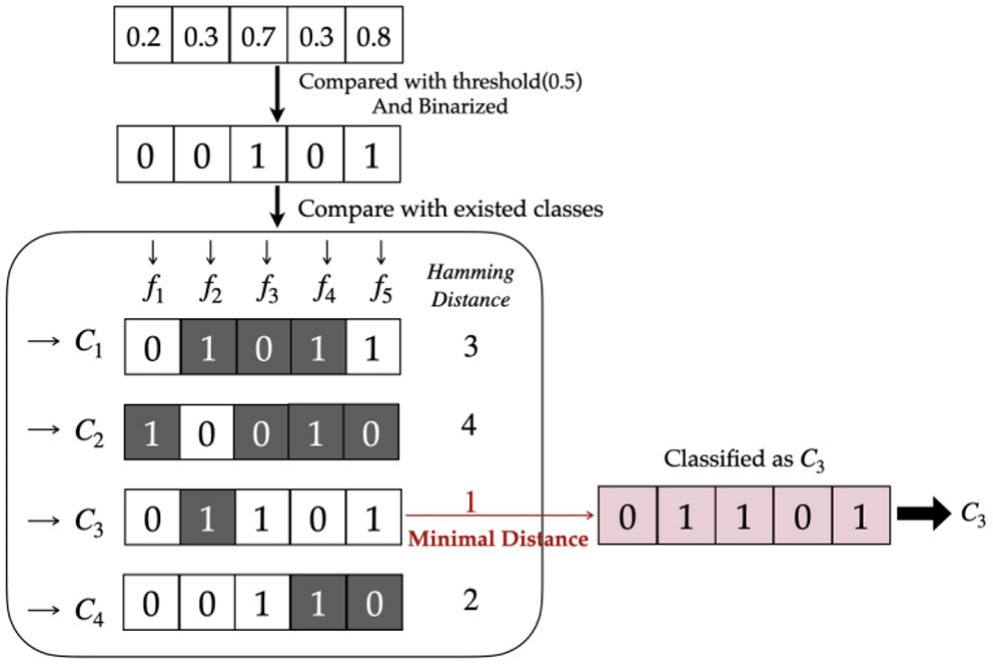
Hard decision with Hamming distance.

**Figure 4:**
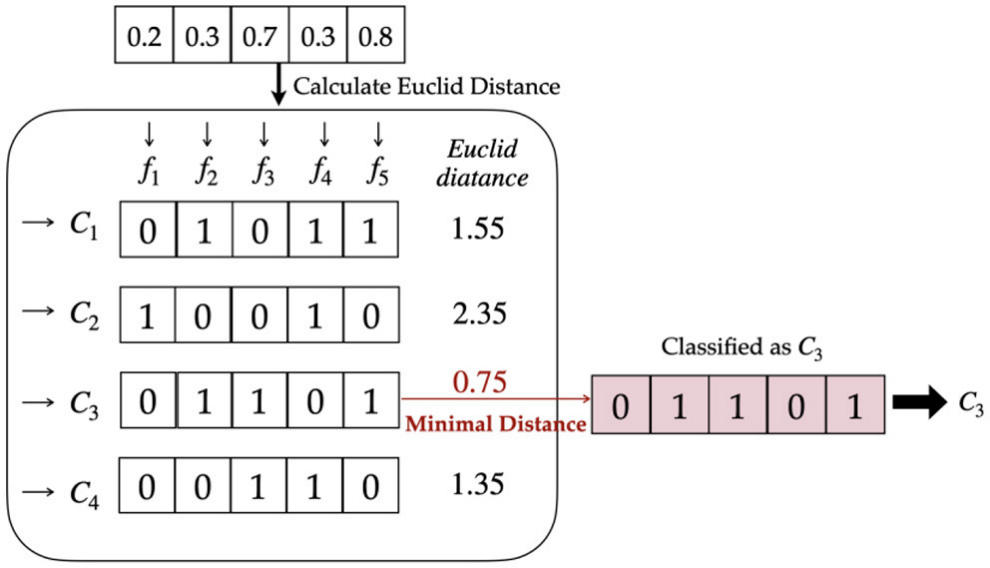
Soft decision with Euclidean distance.

1. Coding Scheme
  Considering that the number of classes is 8 for femoral components and 9 for tibial components, the coding length is set to approximately 16, and the model outputs are binarized with 0.5 as the threshold and then mapped to classes based on the minimum Hamming distance.
  The first option is a (15, 11) Hamming code with a code length of 15 and 11 message bits. There is a computationally high expense in selecting 8 and 9 codewords for the femoral and tibial components, respectively, among the total of 2^11^ codewords if we adopt an exhaustive method. Table 1 describes an algorithm for the automatic selection of codewords that guarantees that the minimum distance is maximized.
  A Hadamard code with a 16-bit code length is another optional coding scheme, and the Hamming distance between any two codewords is constant at 8. Therefore, we mainly focus on whether the distribution of samples is balanced when selecting codewords. The experimental results in Section Results verify that the Hadamard code performs better in this task.

2) Coding Length
  Coding length is also a crucial factor for performance. In general, a code with longer length means a stronger capability of correcting errors, but it also requires more binary classifiers. If the training difficulty of binary tasks increases after decomposition, the final accuracy decreases. In addition, the distribution of samples observably influences the accuracy of the binary model. Therefore, when we choose the coding length, we focus on not only the minimum distance of codewords but also the distribution of samples after decomposition.
  Here, a Hadamard code is employed as the coding scheme, and the optimal code length is discussed with other modules fixed.
  For the Hadamard code, as the code length increases from *n* to 2*n*, the minimum distance increases by n/2 bits, and the capability of correcting errors increases by *n*/4 bits. Only if the additional binary classifiers with incorrect prediction are less than *n*/4 is the increase in code length effective. Intuitively, this requires that the accuracy of each binary classifier be greater than 75%, which is a determining factor for choosing a 16-bit Hadamard code in Section Result.

3) Decision Rules
  The decision rules significantly enhance classifying performance if selected properly. Hard or soft decisions are the first option to be considered. A soft decision uses the output values of the model directly in the following calculation, while a hard decision binarizes the model outputs with a threshold of 0.5.
  Hamming distance is accordingly applied together with a hard decision to indicate the difference between output and classes. Figure 3 elaborates the detailed mapping procedure.
  Considering the effect of the imbalanced dataset, hard decisions can be improved by designing a new threshold. When the sum of instances in one class overwhelms the other, the threshold can be revised to ${\frac{N_{+}}{N}}_{-}$, where *N*_+_ represents the number of positive classes and *N*_−_ represents the number of negative classes (23).
  When the model outputs are classified by the hard decision rule, output values of 0.1 and 0.2, for instance, may both be binarized to 0, but 0.2 has a larger confidence of the positive class than 0.1. Therefore, soft decisions maintain the original probability and retain more information than hard decisions.
  If a soft decision is employed, the Euclidean distance is calculated to select the class with minimal distance as the prediction. The complete process is shown in Figure 4.
  The Euclidean distance may overwhelm the difference when the output is far different from the label. For example, there are two outputs of 0.8 and 0.9 for negative samples. The Euclidean distance of the latter is larger than that of the former; however, they are both quite wrong. In fact, the region we need to focus on is the difference at approximately 0.5, where the binary classifier has insufficient confidence to classify the sample into either of the classes. When the difference is too large or too small, the distance can be almost saturated. Therefore, a sigmoid-based distance is proposed to solve this problem. It is defined in Equation (5), where *p* is the output and *label* means the real class.
  *d*(*p, label*) = 1/(1+*e*^−(|*p*−*label*|−0.5) ×10^) (5)

**Table 1:** Automatic codewords selection procedure.

Target: a set of codewords S Require: the number of codewords needed N 1.Initialize the weight matrix: w = [0, 3, 4, 5, 6, 7, 8, 9, 10, 11, 12, 15] Index:i = 11 Minimum distance: dist_min_ = w[i]. 2.Add the codeword consisting of all 0s into S, the other codewords compose a candidate set O. 3.Let C be the last element of S, update O: 1)Delete the codeword that the distance between it and C is smaller than dist_min_. 2)Move the first element of updated O to S. 4.Judge whether the number of elements in S is not smaller than N. If yes, jump to step 6; otherwise, jump to step 5 5.Judge whether O is empty. If yes, i-, update the minimum distance dist_min_ = w[i], jump to step 2; otherwise, jump to step 3. 6.End the selection for codewords.

## Results

We obtained 308 valid samples in total consisting of 68 males and 240 females, which were split into a training set, validation set and testing set in a ratio of 4:1:1. Each sample was made up of preoperative X-ray films of the knees and other basic information, i.e., weight and height. The labels to predict included the femoral and tibial prosthesis size. The prediction model attempts to predict the size of both components used in TKA at the same time.

The metric that evaluates the model performance is defined as the ratio of correct predictions. In real scenarios for specific patients, at least three adjacent prosthesis sizes are usually provided for doctors before TKA surgery. For this reason, we suggest that if a label lies in the top three possible classes, it will be regarded as a correct prediction in our experiments.

### Data Collection and Analysis

**Figure 5:**
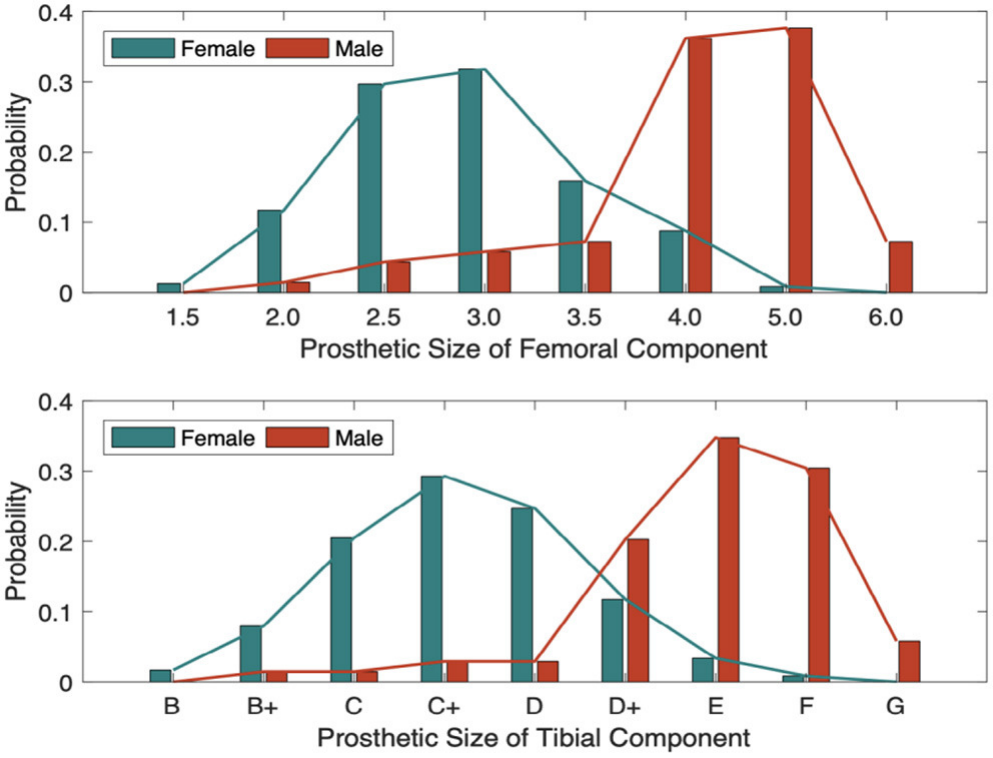
The distribution of prosthetic size with different genders.

The statistical characteristics of the features and labels are further explored, and the results correspond to related medical knowledge of TKA. The distributions of prosthetic size by gender are shown in the histogram of Figure 5. The conclusion can be drawn that the distributions of both labels resemble the normal distribution, and that the basic information, i.e., gender, height, and weight, have a significant impact on the size of both the femoral and tibial components. On the other hand, both the small number of data samples and the uneven distribution of labels are adverse factors for building precise prediction models.

The correlation coefficient *r* between features and labels is further calculated, and a *t*-test is conducted with Equations (6) and (5) to further provide confidence in the correlation. The results are shown in Tables 2, 3.


(5) $$\begin{array}{r} r=E\left(x-E\left(x\right)\right)E\left(y-E\left(y\right)\right)\;/\sqrt{Var\left(x\right)Var\left(y\right)} \end{array}$$

(6) $$\begin{array}{r} t=r/\sqrt{\left(1-r^{2}\right)/\left(n-2\right)} \end{array}$$

According to the statistical characteristics of the *t*-value, if the practical *t*-value is greater than 2.601, there is 99% confidence that the labels are correlated with features. Thus, with an error risk of less than 1%, we can claim that basic information could influence component size. This provides an analytical basis for measuring the effectiveness of prediction algorithms.

**Table 2:** *t*-test value.

** 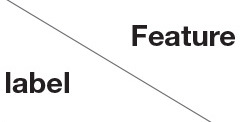 **	**Gender**	**Height**	**Weight**
Femoral	13.65	14.36	7.943
Tibial	14.24	14.77	8.331

**Table 3:** Correlation coefficient.

** 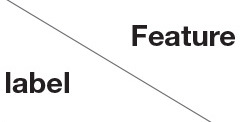 **	**Gender**	**Height**	**Weight**
Femoral	0.6151	0.6345	0.4134
Tibial	0.6314	0.6452	0.4300

### Experiment Results for the Baseline Model

We first show the experimental results from the baseline prediction system. To verify the effect of the ECOC-based model, we compare its experimental results with the baseline model.

We experimented on the baseline model described in Figure 3, and optimizations were implemented in the basic version.

The baseline model was first trained with raw radiographs through the original ResNet18, and the accuracy was 70.59% for femoral components and 68.72% for tibial components.

**Figure 6:**
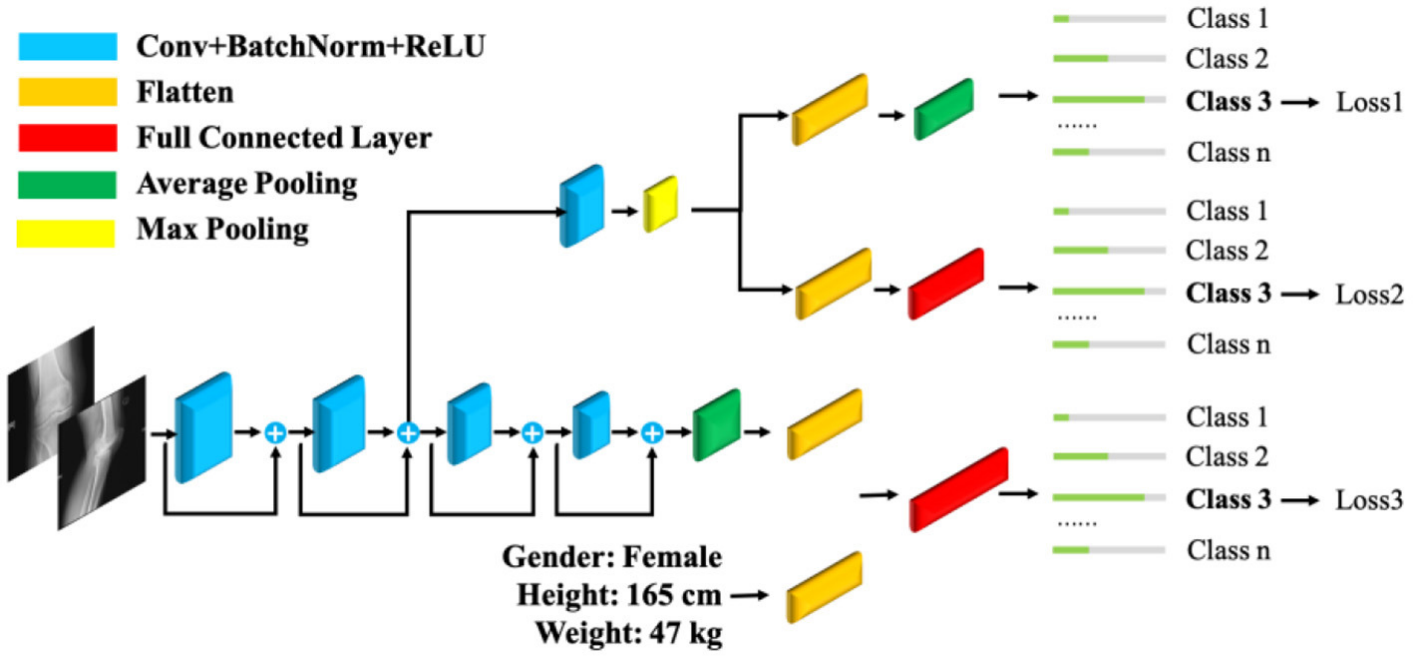
Fine-grained ResNet18 with multimodal data.

To optimize this model, preprocessed radiographs were acquired as substitutive input. Physical information was added to form multimodal data together with the feature maps of the radiographs. For the loss function, the refined focal loss function in Equation (4) was employed. Then, the structure of the network was modified based on inspiration from a fine-grained recognition task. The modified model is illustrated in Figure 6.

Finally, transfer learning was applied, and a fine-grained ResNet18 using a refined loss function, with the addition of transfer learning and multimodal data, was modeled. The optimized baseline model eventually achieved an accuracy of 84.31% for both components. As reported in (24), the accuracy of prediction by experienced doctors through preoperative X-ray images and CT scans is 84% at best. Our system achieves the same level of accuracy at a lower cost.

### Experiment Results for the ECOC-Based Model

The ECOC-based model was constructed on the optimized baseline model. As discussed in Section Methods, appropriate parameters and decision rules were tested and selected.

**Figure 7:**
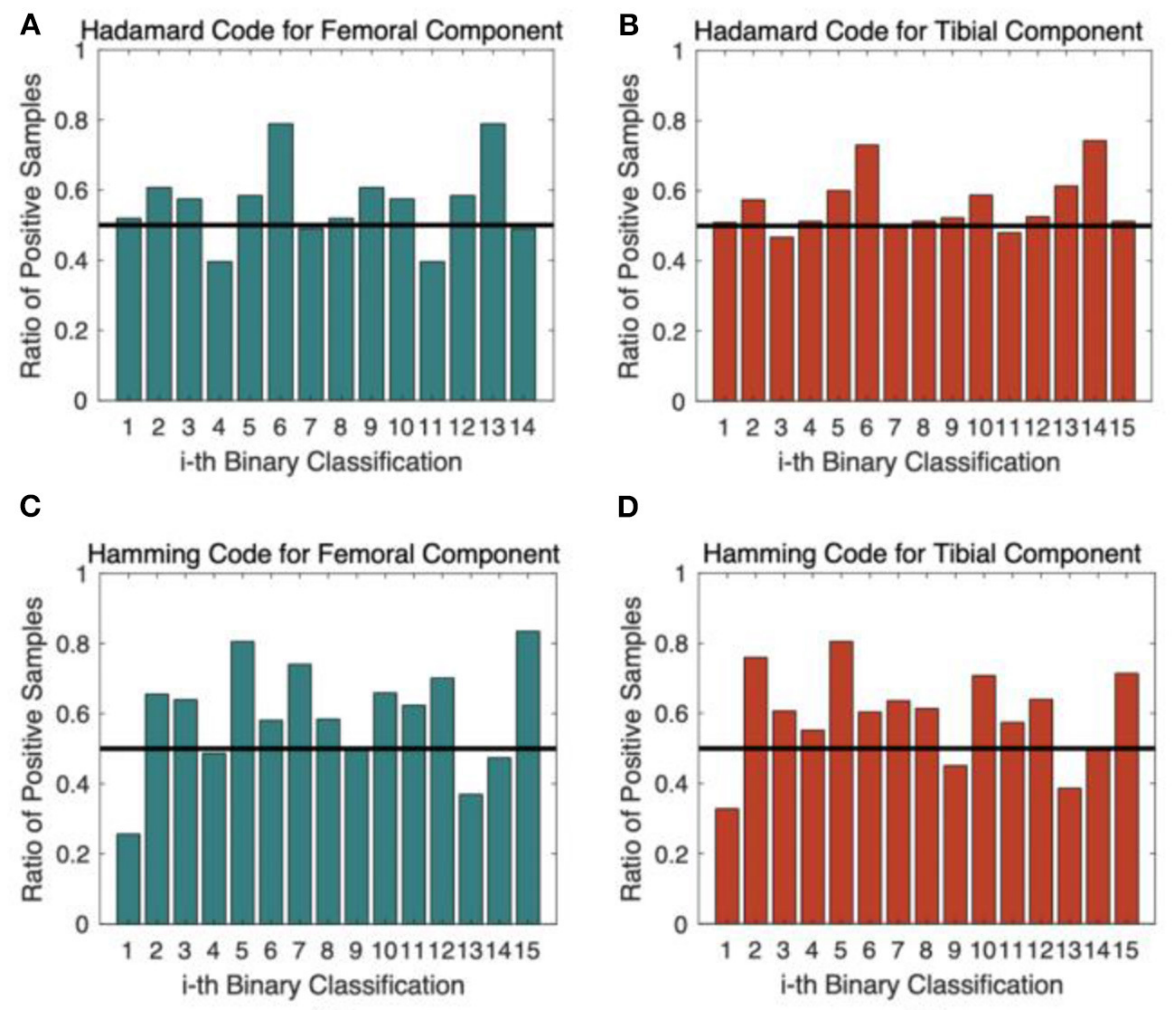
The ratio of positive samples for each binary classifier. **(A)** Hadamard Code for Femoral Component Prediction; **(B)** Hadamard Code for Tibial Component Prediction; **(C)** Hamming Code for Femoral Component Prediction; **(D)** Hamming Code for Tibial Component Prediction.

Figure 7 shows the ratio of positive samples of each binary classifier under different coding schemes. The distribution for the Hadamard code in Figures 7A,B was more balanced than that of the Hamming code in Figures 7C,D because the ratio of positive samples was closer to 50%, which was beneficial to achieving better performance. The experimental results verify that the Hadamard code outperforms the Hamming code by more than 2%. The computational complexity of selecting codewords is also a disadvantage for Hamming code, so we finally applied the Hadamard code as the code scheme.

**Figure 8:**
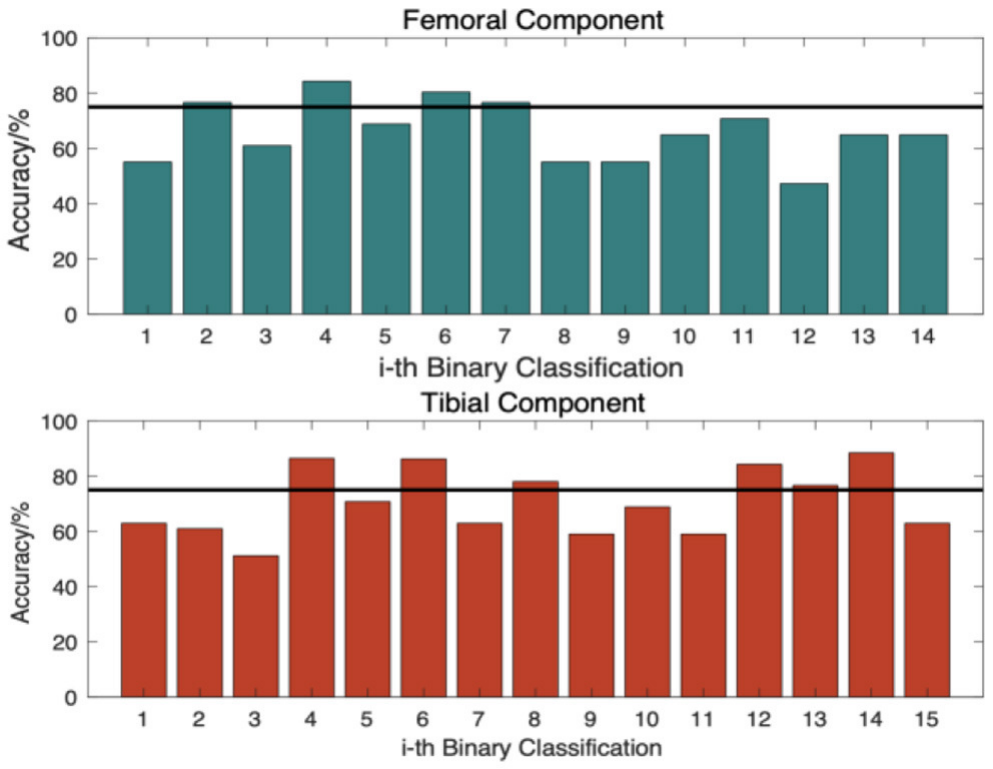
Accuracy of each binary classifier.

Figure 8 shows the accuracies of binary models for a Hadamard code with a 16-bit code length, most of which were smaller than 75%. As discussed in Section Methods, such evidence shows that more than n/4 bit errors appear, so the increase in code length does not bring a distinct enhancement to performance. Thus, a code length of 16 bits is the optimized selection for the system.

**Figure 9:**
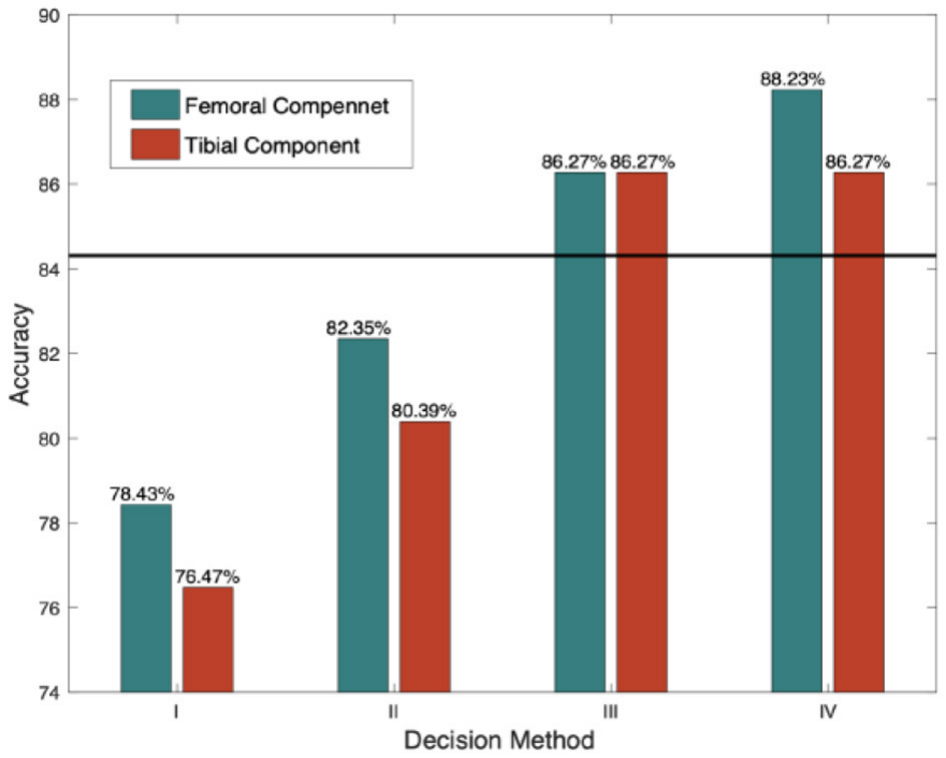
Accuracies in different decision rules.

Performances with different decision rules are illustrated in Figure 9. Method-I refers to hard decision rules, and in Method- II, the threshold was revised. The Hamming distance was adopted as the distance metric in these two methods. Method III and IV utilized soft decision rules with Euclidean and sigmoid-based distances, respectively. The black line represents the best performance of the baseline model. It also represents the accuracy of manual measurements conducted by common surgeons.

We can see that Method IV with sigmoid-based distance achieves the best performance because it focuses on a reasonable distance region. Another reason why this model can obtain higher accuracy is that the distribution of labels after decomposition is more balanced than that of the multiclass model.

## Discussion

Accurate prosthesis matching is a key factor to gain good clinical function and postoperative satisfaction after total knee arthroplasty (4). On the opposite, complications such as postoperative knee pain, aseptic loosening, and poly wearing might happen due to overall or mismathcing of prosthesis (25).

Unfortnately, the selection of prostheses depends on the surgeon's intraoperative experience, which has great subjectivity and long learning curve. Preoperative templating is helpful prosthesis matching, and also can simplify the prosthesis preparation, which effectively reduce medical costs, improve the work efficiency of the operating room, and obtain good health economic benefits (26).

Preoperative templating is highly dependent on quality of X-ray, and the inherent limitation of X-ray magnification has a significant impact on the prediction of prosthesis, which is difficult to completely eliminate even if with a metal ball as a marker (3, 27, 28).

With the continuous maturity and development of image recognition and deep learning, we have reason to believe that the accuracy size of prosthesis might be predict before surgery by artificial intelligence technique with non-marker X-ray (29–31).

The performances of the three primary models in our study are summarized in Table 4. By providing the model with the capability of correcting errors, the ECOC-based algorithm reaches accuracies of 88.23 and 86.27%, outperforming the optimized baseline model by 4% for femoral components and 2% for tibial components, which is better than the average accuracies of experienced doctors. The practical value of this research is further confirmed through verification experiments.

**Table 4:** Accuracy metrics for different cases.

**Models**	**Femoral (%)**	**Tibial (%)**
Baseline model	70.59	68.72
Optimized baseline model	84.31	84.31
ECOC-based model	**88.23**	**86.27**

*The bold values are best performances of proposed models*.

This article proposes a novel software system for automatic prediction of prosthetic sizes in TKA based on a CNN, with patient gender, BMI and non-marked X-ray. Many optimizations are implemented in the loss function, model structure and transfer learning to solve the problems of data deficiency and uneven label distribution. To further boost the performance, we introduce ECOC to the classification algorithm, which decomposes the original multiclass problem into several binary subproblems and then trains the corresponding binary classifiers jointly. The ECOC-based prediction system exceeds the direct multiclass classifier in terms of prediction accuracy and surpasses the average level of manual measurements conducted by surgeons.

There are limitations to be addressed: (1) From the methodological point of view, the amount of observed samples is still relatively deficient to train a perfect model. (2) The model training process is based on the experts' intra-operative decisions; thus, the prediction accuracy has an upper bound of the ground truth, that is, the surgeon expertise. (3) For the concern of expense, the prediction model only based on X-ray images and BMI information. If the information of other medical images is taken into account, the performance may be further enhanced.

To conclude, our project proposes an intelligent prediction system that does not depend on the quality of X-rays or require reference balls. It achieves acceptable accuracy from a small existing dataset. This system can offer optimal TKA component size suggestions to doctors, especially those with limited experience. It can also help reduce labor and sterilization costs.

While the research conducted on real medical diagnostic data sheds light on how to improve the performance of learning with small datasets, it also gives impetus to the application of AI-aided tools in the medical sector. Our work may contribute to the development of intelligent medicine for the 5G or the coming 6G era.

## Data Availability Statement

The original contributions presented in the study are included in the article/supplementary material, further inquiries can be directed to the corresponding author/s.

## Ethics Statement

Written informed consent was not obtained from the individual(s) for the publication of any potentially identifiable images or data included in this article.

## Author Contributions

YY: conceptualization, methodology, validation, formal analysis, investigation, software, and writing—original draft. QG: methodology, investigation, software, and writing—editing. MZ: conceptualization, validation, investigation, resources, data curation, and writing—review. DL: conceptualization and writing—review and editing, supervision, project administration, and funding acquisition. HT: conceptualization, validation, investigation, resources, data curation, and writing—review. All authors contributed to the article and approved the submitted version.

## Funding

This study was funded by National Key R&D Program of China under Award Numbers 2020YFB1807802, 2016ZX03001018-005 and Peking University Medicine Fund of Fostering Young Scholars' Scientific & Technological Innovation under Award Number BMU2021PYB034.

## Conflict of Interest

The authors declare that the research was conducted in the absence of any commercial or financial relationships that could be construed as a potential conflict of interest.

## Publisher's Note

All claims expressed in this article are solely those of the authors and do not necessarily represent those of their affiliated organizations, or those of the publisher, the editors and the reviewers. Any product that may be evaluated in this article, or claim that may be made by its manufacturer, is not guaranteed or endorsed by the publisher.
